# A DNA Electrochemical Sensor via Terminal Protection of Small-Molecule-Linked DNA for Highly Sensitive Protein Detection

**DOI:** 10.3390/bios11110451

**Published:** 2021-11-13

**Authors:** Ping Ouyang, Chenxin Fang, Jialun Han, Jingjing Zhang, Yuxing Yang, Yang Qing, Yubing Chen, Wenyan Shang, Jie Du

**Affiliations:** State Key Laboratory of Marine Resource Utilization in South China Sea, College of Materials Science and Engineering, Hainan University, Haikou 570228, China; oypoypoypylyz@163.com (P.O.); fcx19962021@163.com (C.F.); jialun_han@126.com (J.H.); zhangjingjingaoxue@163.com (J.Z.); thea0208@163.com (Y.Y.); 15859267122@139.com (Y.Q.); robynnchan@foxmail.com (Y.C.); 15501862735@163.com (W.S.)

**Keywords:** terminal protection, electrochemical DNA biosensor, streptavidin, Exonuclease III, small-molecule-linked DNA

## Abstract

The qualitative and quantitative determination of marker protein is of great significance in the life sciences and in medicine. Here, we developed an electrochemical DNA biosensor for protein detection based on DNA self-assembly and the terminal protecting effects of small-molecule-linked DNA. This strategy is demonstrated using the small molecule biotin and its receptor protein streptavidin (SA). We immobilized DNA with a designed structure and sequence on the surface of the gold electrode, and we named it M1-Biotin DNA. M1-Biotin DNA selectively combines with SA to generate M1-Biotin-SA DNA and protects M1-Biotin DNA from digestion by EXO III; therefore, M1-Biotin DNA remains intact on the electrode surface. M1-Biotin-SA DNA was modified with methylene blue (MB); the MB reporter molecule is located near the surface of the gold electrode, which generates a substantial electrochemical signal during the detection of SA. Through this strategy, we can exploit the presence or absence of an electrochemical signal to provide qualitative target protein determination as well as the strength of the electrochemical signal to quantitatively analyze the target protein concentration. This strategy has been proven to be used for the quantitative analysis of the interaction between biotin and streptavidin (SA). Under optimal conditions, the detection limit of the proposed biosensor is as low as 18.8 pM, and the linear range is from 0.5 nM to 5 μM, showing high sensitivity. The detection ability of this DNA biosensor in complex serum samples has also been studied. At the same time, we detected the folate receptor (FR) to confirm that this strategy can be used to detect other proteins. Therefore, this electrochemical DNA biosensor provides a sensitive, low-cost, and fast target protein detection platform, which may provide a reliable and powerful tool for early disease diagnosis.

## 1. Introduction

Protein is one of the typical biomarkers [1]. Highly sensitive detection of protein is necessary in early diagnosis and disease treatment [2]. In this work, we used the biotin-streptavidin system as a template system to develop an electrochemical DNA biosensor that uses small molecule–protein molecule interactions to detect target proteins. In recent years, research and development of the interaction between small molecules, proteins, and other biological macromolecules has attracted increasing attention, which is the basis of signal transduction, energy transfer, metabolism, and functional regulation [3]. Exploiting the interaction of small molecules with proteins and other biological macromolecules to enable the specific binding of small molecules to proteins with strong affinity is essential in the fields of biogenetics and macromolecular detection [4,5]. The specific binding of some small molecules to proteins destroys some of the functions of the protein receptor. As a result, this interaction has potential applications in the fields of drug development, molecular therapy, and biomedicine [6]. In addition, the interaction of small molecules with proteins can also be used to detect proteins [7,8,9,10]. At present, small-molecule-linked DNA is an effective tool used to detect the interaction between small molecules and their protein receptors [11]. 

Recently, Li [12] et al. discovered that when a small molecule binds to its target protein, the DNA that connects the small molecule is protected from being digested by Exonuclease III (Exo III). When a small molecule binds to its target protein, a strong binding force between the small molecule and receptor protein exists and at the same time, due to strong steric hindrance, the protein prevents EXO III from approaching and cleaving the DNA phosphodiester bond. As a result, the protein provides protection to the 3′ end of the DNA linked using this small molecule, and prevents hydrolyzation by EXO III [13,14]. Furthermore, based on the terminal protection effect and EXO III-catalyzed dsDNA digestion, Zhang [15] and colleagues developed a new FRET model based on quantum dots and tungsten disulfide nanosheets that act as energy donor–acceptor pairs. This model has been successfully applied for the detection of protein via the small molecule terminal protection method of DNA ligation.

In this work, based on the small molecule–target protein interaction that protects the 3′ end of DNA linked using a small molecule, we developed an electrochemical DNA biosensor for the detection of proteins. Streptavidin (SA) is a protein secreted by Streptomyces avidinii during the growth process [16]. SA has a high affinity with biotin [17]. SA is composed of four identical peptide chains, each of which can be combined with a biotin molecule. The interaction between them is non covalent, with strong affinity, good stability and strong specificity. SA is used in many aspects, such as biochemical sensors and nano biotechnology [18]. Therefore, we demonstrated this strategy by using a small molecule biotin and its receptor protein SA as a model example [19]. We immobilized a DNA with a designed structure and sequence on the surface of the gold electrode, and we named it M1-Biotin DNA. M1-Biotin DNA specifically combines with SA to generate M1-Biotin-SA DNA and protects M1-Biotin DNA from digestion by EXO III. Therefore, M1-Biotin DNA remains on the electrode surface. The presence of methylene blue (MB)-modified M1-Biotin-SA DNA and the proximity of the MB reporter molecule to the gold electrode surface results in the generation of a large electrochemical detection signal [20,21,22,23]. Therefore, the presence or absence of an electrochemical signal is used to qualitatively analyze SA and the strength of the electrochemical signal is used to quantitatively analyze the concentration of SA [24]. This strategy transforms protein detection into the detection of deoxyribonucleic acid [25]. We speculate that it can detect protein molecules that specifically interact with small molecules, such as folate receptors (FR-FA) [12].Folate receptor alpha (FR-α) [26] has been identified as a potential marker of ovarian cancer for diagnostic and therapeutic purposes, based on its overexpression in serous epithelial ovarian cancer. Compared with complex protein analysis methods that usually involve separation and purification steps, this strategy is sensitive, simple, inexpensive, and rapid [27,28,29]. Based on these preliminary studies, the method is expected to provide a new idea for the detection of proteins.

## 2. Materials and Methods

### 2.1. Reagents and Materials

#### 2.1.1. Materials

All oligonucleotides (Table 1) were synthesized and purified by Sangon Biotech Inc. (Shanghai, China). The 5′ end of B1-DNA was thiolated with –(CH_2_)_6_SH– as a spacer and the other (3′ end) was modified with biotin. S1-DNA and S2-DNA were modified with MB at the 3′ end and used as signal probes.

6-Mercapto-1 hexanol (MCH), potassium chloride (KCl), sodium chloride (NaCl), magnesium chloride (MgCl_2_), potassium ferricyanide (K_3_Fe(CN)_6_), and potassium ferrocyanide (K_4_Fe(CN)_6_) were purchased from Aladdin Bio-Chem Technology Co., Ltd. (Shanghai, China). EXO III was purchased from New England Biolabs Co. Ltd. (Guangzhou, China). Sulfuric acid was purchased from Xilong Scientific Co., Ltd. (Guangzhou, China). Streptavidin was purchased from Sigma-Aldrich (St. Louis, MO, USA).

We used ultrapure water (Dongsheng Biotech Co., Ltd., Resistivity 18.2 MΩ) to prepare all solutions. Additionally, according to the requirements, the solutions are stored at 4 °C or −20 °C, respectively.The DNA hybridization buffers contained 50 mM NaCl, 10 mM MgCl_2_, and 10 mM Tris–HCl (pH 7.4). The wash buffer contained 100 mM NaCl and 10 mM Tris–HCl (pH 7.4). The buffer used for the square wave voltammetry (SWV) analyses contained 100 mM NaCl, 20 mM MgCl_2_, and 10 mM Tris–HCl (pH 7.4).

#### 2.1.2. Electrochemical Measurements

We carried out all the electrochemical measurements on the CS350H electrochemical workstation (Wuhan CorrTest Instruments Corp., Ltd., Wuhan, Hubei, China). In all tests including SWV, CV, and EIS, a conventional three-electrode configuration was used. We used gold electrode as the working electrode, platinum wire electrode as the auxiliary electrode, and Ag/AgCl as the reference electrode. The electrochemical impedance spectroscopy (EIS) of the gold electrode was tested in a 0.1 M KCl solution containing 5 mM [Fe(CN)_6_]^3−/4−^. The conditions for electrochemical impedance spectroscopy (EIS) are: the frequency range is 1 Hz-100 kHz, and the potential is 0.214 V. In the same mixed solution, a cyclic voltammetry (CV) test was performed. The conditions for cyclic voltammetry (CV) are: a scan rate of 50 mV/s and a potential window of -0.2 V to 0.6 V. The interface assembly process of DNA molecules is usually characterized by CV and EIS.

The square wave voltammetry (SWV) measurement was carried out in 0.1M NaCl, 20 mM MgCl_2_ and 10 mM Tris-HCl (pH 7.4). The scanning potential of SWV is from −0.4 V to 0 V, the potential increment is 1mM, the frequency is 15 Hz, and the amplitude is 25 mV. The calculation formula is as follows: signal difference (ΔI) = I_M_ − I_0_, where I_M_ is the peak current of MB when there is a target, and I_0_ is the peak current when there is no target, and it is also the peak current of the baseline.

### 2.2. Experimental Procedures

#### 2.2.1. Sensor Preparation

We used 1.0 μm, 0.3 μm, and 0.05 μm α-Al_2_O_3_ powder water slurry to polish the gold electrode on the polishing microcloth. The gold electrode should be ground at least 30 times in the α-Al_2_O_3_ powder water slurry of each particle size. Then, the gold electrodes were sonicated in deionized water, ethanol, and deionized water for 5 min and rinsed with deionized water. Finally, an electrochemical CV scan (scanning potential: −0.4 V–1.6 V) was performed in 0.5M dilute sulfuric acid until a good oxidation and reduction peak of the gold electrode appeared, and a stable cyclic voltammogram (CV) curve was obtained.

The electrochemical DNA sensor fabrication involved several steps. First, B1-DNA was dissolved into a hybridization buffer (containing 1 mM TCEP) to configure a 1.5 µM B1-DNA solution. For B1-DNA immobilization, a 6 μL B1-DNA droplet was dropped onto the electrochemically activated gold electrode and incubated overnight at room temperature. As a result, B1-DNA was immobilized on the gold electrode via thiol–gold interactions. The next day, the modified electrodes were immersed in a 2 mM MCH solution for one hour to remove the non-specific adsorbed DNA. The signal probes were prepared by mixing equal volumes of 5 μM S1-DNA and 3 μM S2-DNA dissolved in the hybridization buffer, then heating the mixed signal probes to 90 °C for 5 min and slowly cooling them to room temperature over at least 2 h. For DNA self-assembly, 6 μL signal probe droplets were dropped onto the B1-DNA-modified gold electrode surface and left for at least two hours at room temperature to achieve adequate hybridization to form M1-Biotin DNA. The modified electrode was thoroughly rinsed with wash buffer. At this point, the electrochemical DNA sensor was fully constructed.

#### 2.2.2. SA Detection Procedure

The M1-Biotin DNA-modified electrodes were immersed in buffer (20 mM Tris-HCl, 100 mM NaCl, 20 mM MgCl_2_) containing SA and incubated at 37 °C for 30 min. The M1-Biotin DNA modified electrode with fully bound SA was then immersed into a 1 U/μL (buffer = 1 × NEBuffer) solution of EXO III and allowed to fully digest. The modified electrodes were then immersed in an electrolytic cell for testing.

## 3. Results and Discussion

### 3.1. Detection Strategy

**Figure 1:**
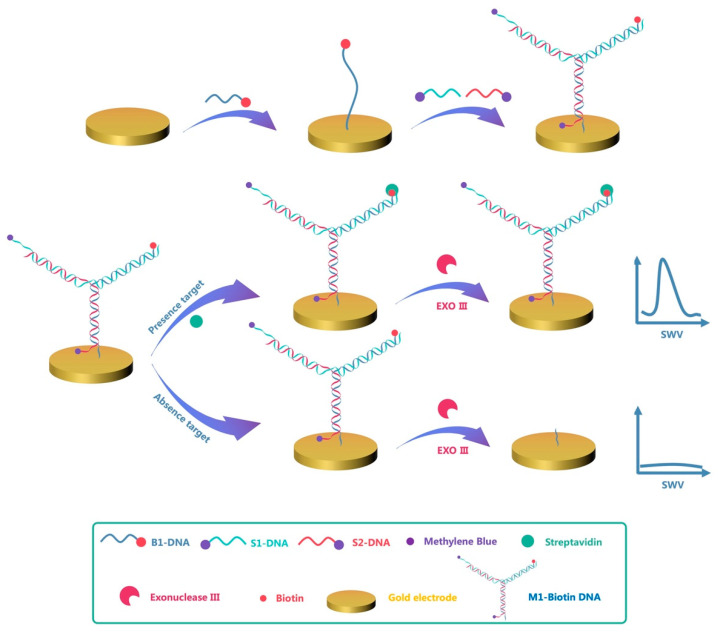
Design and signal mechanism of the electrochemical DNA sensor based on terminal protection. In the absence of SA, M1-Biotin DNA gradually hydrolyzes from the 3′ end of the modified biotin under the action of EXO III until no M1-Biotin DNA remains on the electrode. When M1-Biotin DNA is digested by EXO III, the S1-DNA and S2-DNA modified with the reporter molecule MB is removed from the electrode, meaning only a small electrochemical signal is detected. In the presence of SA, M1-Biotin DNA specifically binds with SA to generate M1-Biotin-SA DNA, which protects M1-Biotin DNA from EXO III digestion, meaning M1-Biotin DNA remains on the electrode surface. Due to the presence of MB on M1-Biotin-SA DNA and the proximity of the MB reporter molecule to the gold electrode surface, a large electrochemical detection signal is generated.

Figure 1 shows the operating principle of the electrochemical DNA sensor based on terminal protection. B1-DNA is modified with a sulfhydryl group ((−CH_2_)_6_SH) at the 5′ end and self-assembled on a gold electrode via gold–thiol bonds, whereas the 3′ end of B1-DNA is modified with a small molecular recognition compound biotin. Next, the gold electrode modified with B1-DNA was passivated using MCH to remove non-specifically adsorbed DNA. M1-Biotin DNA was generated by hybridization of S1-DNA and S2-DNA modified with MB at the 3′ end with B1-DNA. In order to protect the MB reporter molecule from EXO III digestion, the 3′ ends of S1-DNA and S2-DNA were extended by five nucleotide bases relative to the M1-Biotin DNA duplex. EXO III catalyzes the gradual removal of single nucleotides from the 3′ end of dsDNA along the DNA strand. Since the 3′ends of S1-DNA and S2-DNA do not meet the working conditions of EXO III, they cannot be hydrolyzed. Therefore, in the presence of EXO III, M1-Biotin DNA will only gradually hydrolyze from the biotin-modified 3′end, which is the 3′end of B1-DNA, until the B1-DNA is depleted on the electrode. When the B1-DNA is digested by EXO III, the S1-DNA and S2-DNA modified with the reporter molecule MB are removed from the electrode, meaning only a small electrochemical detection signal is produced.

However, by adding SA to the test solution, M1-Biotin DNA specifically binds to SA to generate M1-Biotin-SA DNA and protects the M1-Biotin DNA from EXO III digestion; therefore, M1-Biotin DNA remains on the electrode surface. Due to the presence of MB on M1-Biotin-SA DNA and the proximity of the MB reporter molecule to the gold electrode surface, a large electrochemical detection signal is generated. The intensity of the electrochemical signal is related to the concentration of SA. Based on this, a sensitive electrochemical DNA sensor for the specific detection of protein has been developed.

### 3.2. Modified Electrode Characterization

**Figure 2:**
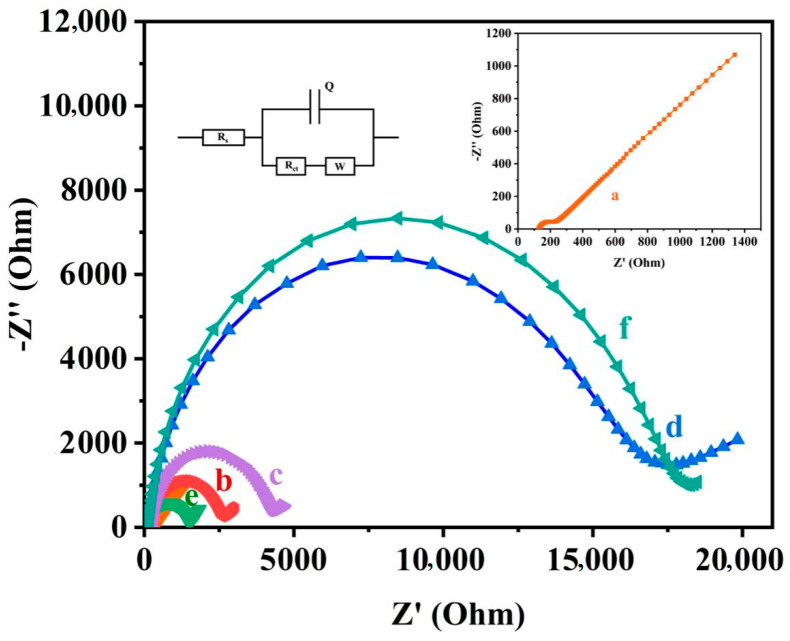
Nyquist plots at different stages of the modification process: (**a**) bare gold electrode; (**b**) B1-DNA, (**c**) M1-Biotin DNA, (**d**) M1-Biotin-SA DNA-modified electrode hydrolyzed by EXO III, (**e**) M1-Biotin DNA-modified electrode hydrolyzed by EXO III, (**f**) M1-Biotin-SA DNA-modified electrode. The data were recorded in the presence of 5 mM [Fe(CN)_6_]^3−/4−^ containing 0.1 mol/L KCl, upon application of a bias potential of 0.214 V a 5 mV alternating voltage was applied in the frequency range 1–100 kHz.

In order to prove the feasibility of this DNA biosensor strategy, we first measured electrochemical impedance spectroscopy (EIS) to characterize the interface characteristics of the electrode surface with different modification states. Figure 2 shows the Nyquist diagram of the sensor platform at different stages. The semicircle in the Nyquist diagram represents the electron transfer limited process, and the diameter of the semicircle represents the interface charge transfer resistance (R_ct_) of the redox probe [Fe(CN)_6_]^3−/4−^ at a certain potential [30]. The linear part represents the diffusion-limited process.From Figure 2, we can see that the bare gold electrode essentially does not have a semicircular domain (Rct = 100 Ω, curve (a)), which shows the obvious characteristics of the electrochemical diffusion limitation process. After fixing the B1-DNA linked with thiolated small molecules, we can observe a clear semicircle (Rct = 3000 Ω, curve (b)). This is because with the increase in the negative charge on the electrode and the hindering effect of the small molecule Biotin, the redox probe [Fe(CN)_6_]^3−/4−^ is more difficult to transfer on the electrode surface, and the charge transfer resistance (Rct) increases. In addition, the inactivation of B1-DNA in the MCH solution and the hybridization of S1-DNA and S2-DNA on the electrode surface also led to a further increase in the diameter of the semicircle (Rct = 4790 Ω, curve (c)). The double negatively charged phosphoric acid skeleton fixed on the electrode and the MCH modified on the electrode further hinder the charge transfer of [Fe(CN)_6_]^3−/4−^ on the electrode interface, thereby increasing the charge transfer resistance (Rct). The diameter of the semicircle is obviously reduced (Rct = 1500 Ω, curve (e)) with the direct use of EXO III to digest the M1-Biotin DNA probe. This is because the M1-Biotin DNA probe exposes the 3′ end, whereas EXO III can digest double-stranded DNA (dsDNA) from the 3′ end to the 5′ end, and the digested product will leave the electrode. As a result, the charge transfer resistance (Rct) is significantly reduced. However, after the M1-Biotin DNA-modified electrode is treated with target protein (SA), and the diameter of the semicircle is greatly increased (Rct = 19,500 Ω, curve (f)). The reason lies in the combination of streptavidin and Biotin in M1-Biotin DNA. Because the molecular weight of streptavidin (SA) is very large, the charge transfer of [Fe(CN)_6_]^3−/4−^ on the electrode interface has a large steric hindrance, resulting in a sharp increase in charge transfer resistance (Rct). After EXO III digestion (Rct = 18,700 Ω, curve (d)), because some M1-Biotin DNA not bound to SA was digested by EXO III, the diameter of the semicircle was slightly reduced. It is also proved that streptavidin (SA) can protect the 3′ end of the bound M1-biotin DNA from EXO III hydrolysis.

**Figure 3:**
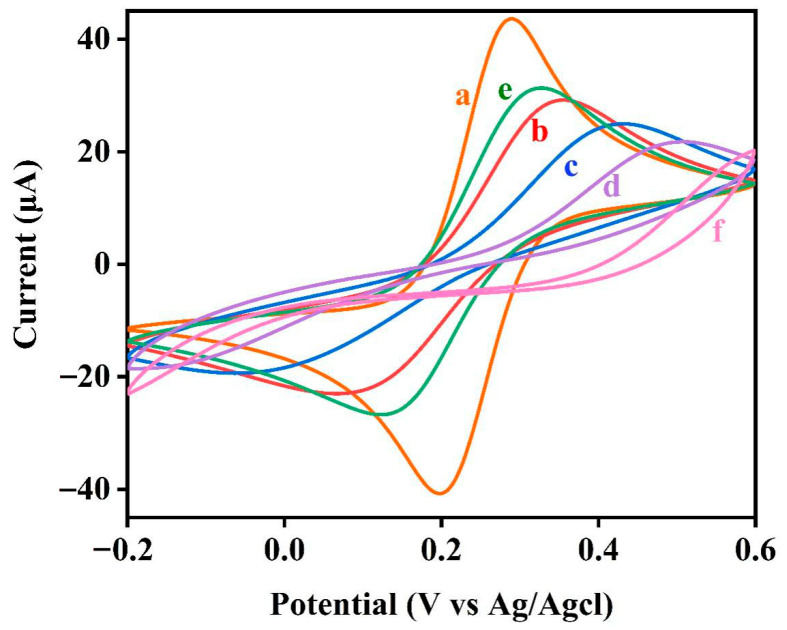
Cyclic voltammograms at different stages of electrode modification: (**a**) bare gold electrode, (**b**) B1-DNA, (**c**) M1-Biotin DNA, (**d**) M1-Biotin-SA DNA-modified electrode hydrolyzed by EXO III, (**e**) M1-Biotin DNA modified electrode hydrolyzed by EXO III, (**f**) M1-Biotin-SA DNA-modified electrode. The data were recorded in the presence of 5mM [Fe(CN)_6_]^3−/4−^ containing 0.1 mol/L KCl by scanning the potential from −0.2 V to 0.6 V at a scan rate of 100 mV/s.

The electrochemical behavior of the electrochemical DNA sensor was also characterized using CV. As shown in Figure 3, the peak current of the B1-DNA-modified electrode (curve b) significantly decreased and the separation between the redox peaks increased compared with the bare gold electrode (curve a). After immersing the modified electrode into MCH and then hybridizing B1-DNA with S1-DNA and S2-DNA (curve c), the peak current decreased further, whereas the separation between the redox peaks increased further. Direct digestion of M1-Biotin DNA using EXO III (curve e) resulted in a significant increase in peak current. However, following treatment of the electrode with the target protein (SA) (curve f), the peak current decreased significantly. The reason for this is that, due to SA having a high molecular weight, the binding of SA and biotin in M1-Biotin DNA substantially hinders the charge transfer process of the redox probe [Fe(CN)_6_]^3−/4−^ at the electrode interface. After digestion with EXO III (curve d), because some M1-Biotin DNA that was not bound to SA was digested by EXO III, the peak current increased but was still at a small value. Using CV and EIS, we have shown that SA protects the 3′ end of M1-Biotin DNA from EXO III hydrolysis.

### 3.3. Detection Feasibility Assay

**Figure 4:**
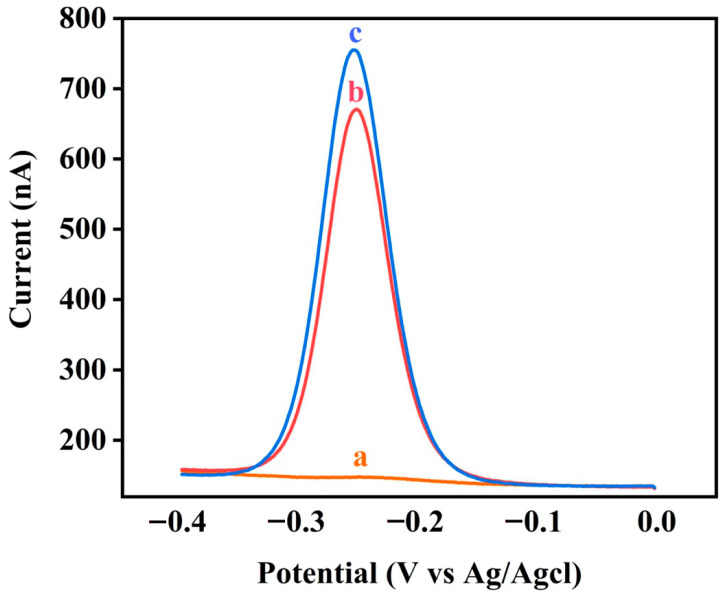
Square wave voltammetry performed using the M1-Biotin DNA-modified electrode in a buffer containing 0.1 M NaCl, 20 mM MgCl_2_ and 10 mM Tris–HCl (pH 7.4), (**a**) without SA (incubated with EXO III), (**b**) containing 5 μM SA (incubated with EXO III), and (**c**) containing only M1-Biotin DNA (not incubated with EXO III).

A simple experiment was performed to demonstrate our hypothesis. Figure 4 shows SWV performed using the M1-Biotin DNA modified electrode in a buffer containing 0.1 M NaCl, 20 mM MgCl2, and 10 mM Tris–HCl (pH 7.4); (a) without SA, (b) containing 5 μM SA, and (c) containing only M1-Biotin DNA. When SA is not present in the solution, a weak electrochemical signal is detected. When SA is added to the solution, a strong electrochemical signal is observed. This indicates that M1-Biotin DNA is digested by EXO III without the protection of SA. Therefore, the proposed strategy effectively detects the interaction between biotin and the target protein SA.

### 3.4. Experimental Parameter Optimization

**Figure 5:**
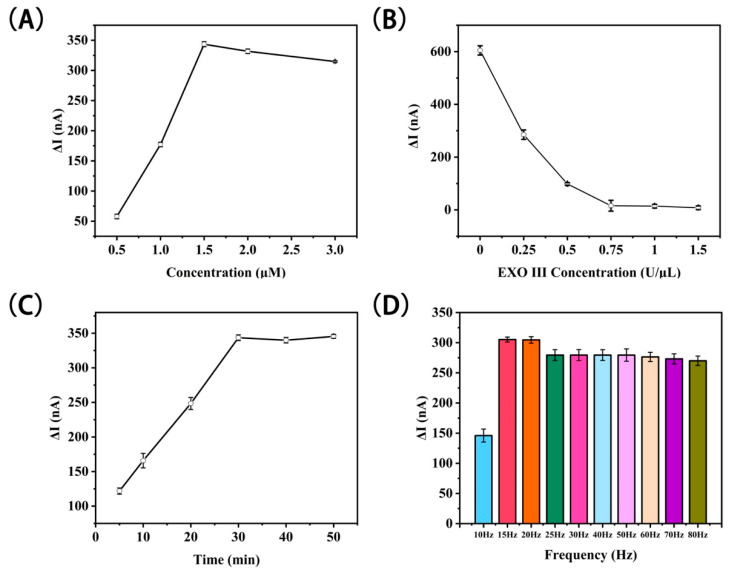
Optimization of the electrochemical DNA sensor based on terminal protection. The effect of (**A**) modified electrode DNA concentration, (**B**) EXO III concentration, (**C**) incubation time of the modified electrode in SA, (**D**) frequency of the electrochemical DNA sensors. Error bars represent the standard deviation of the measurement (n = 3).

To achieve the optimal performance of this electrochemical DNA sensor, we focused on four key parameters, namely DNA concentration, EXO III concentration, reaction time in the presence of SA, and SWV frequency. In order to ensure optimal DNA self-assembly during the experiment, the ratio of B1-DNA, S2-DNA, and S1-DNA was 1:1:1.25. The concentration of B1-DNA is discussed next. In addition to the conditions that need to be optimized, other conditions are maintained at their optimal values in the experiment. The SA concentration used in these experiments was 100 nM. As shown in Figure 5A, the intensity of the SWV signal gradually increased as the DNA concentration increased and eventually converged to a stable plateau at a concentration of 1.5 μM; thereafter, the value slowly decreased. The maximum SWV signal intensity was achieved at a concentration of 1.5 μM. Therefore, to ensure optimal sensor performance a DNA concentration of 1.5 μM was selected.

We immersed the modified electrode (not incubated with SA) in a various concentrations of EXO III for 20 min. As shown in Figure 5B, the SWV signal intensity decreased with increasing EXO III concentration and fell to zero at concentrations of 0.75 U/μL and above. In order for EXO III to adequately digest the DNA present on the electrode, an EXO III concentration of 1 U/μL was used in further experiments.

The incubation time of M1-Biotin DNA in SA also has a significant impact on the implementation of this electrochemical DNA sensor. As shown in Figure 5C, the SWV signal intensity increased with increasing SA incubation time and reached a plateau at an incubation time of 30 min, after which the value slowly decreased. Therefore, an SA incubation period of 30 min was selected.

The SWV frequency also influences the application of the electrochemical DNA sensor. As shown in Figure 5D, the current reached a peak at a frequency of 15 Hz and then plateaued at frequencies of 25 Hz and above. In addition, the SWV signal at 15 Hz is smoother and more stable than at other frequencies; as such, 15 Hz was selected for further experiments.

### 3.5. Detection Performance

**Figure 6:**
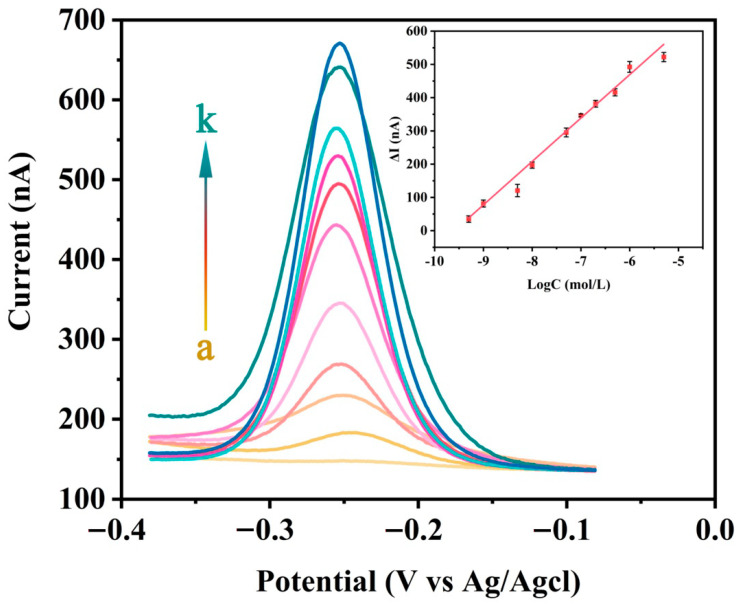
Typical SWV responses of the electrochemical DNA sensors to different concentrations of SA, from bottom to top (a to k): 0 nM, 0.5 nM, 1 nM, 5 nM, 10 nM, 50 nM, 100 nM, 200 nM, 500 nM, 1 μM, and 5 μM in a buffer solution (10 mM Tris–HCl, 100 mM NaCl, 20 mM MgCl_2_, pH 7.4). Logarithmic graph (inset) of ΔI (nA) and target protein SA concentration (0.5 nM to 5 μM). Error bars represent the standard deviation of the measurement (n = 3).

In order to test the sensitivity of this electrochemical DNA sensor, different concentrations of SA were detected under optimal experimental conditions. As shown in Figure 6, as the concentration of SA increases the peak SWV current gradually increases, which indicates that the quantitative analysis signal response is highly concentration-dependent. At SA concentrations between 0.5 nM and 5 μM, the logarithm of the SA concentration is proportional to the SWV peak current. Through linear fitting, the following linear regression equation is obtained:

$$\Delta I(\mathrm{nA})=1243.81+130.45\log C(M),\;R^{2}=0.9828$$

In the formula, ΔI is the difference between the peak current I_M_ and the baseline current I_0_, it is also the difference between the SWV signal intensity in the presence and absence of SA. R is the regression coefficient. C is the concentration of SA (mol/L). Using the formula 3σ/S (σ is the standard deviation of the blank signal and S is the slope of the fitted line in the inset of Figure 6), the limit of detection for SA was calculated to be 18.8 pM. Compared with previously reported methods such as fluorescence spectrophotometry and electrochemical methods, this method has a lower detection limit and a wider SA detection linear range. The detection performances are compared with some previous studies and the results are listed in Table 2.

In order to test the reproducibility of this method, the same concentration of SA was analyzed three times under optimal experimental conditions. We found that the relative standard deviation of the SWV signal intensity (n = 3) is 5.73%, which indicates that this method has good repeatability.

### 3.6. Biosensor Specificity

**Figure 7:**
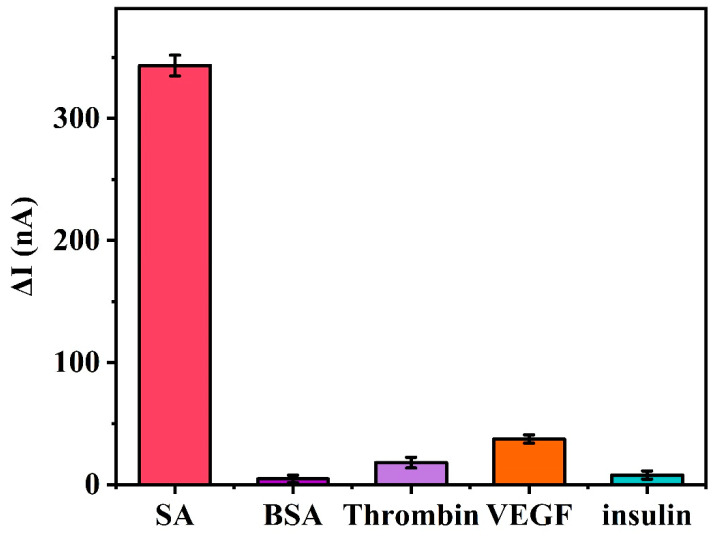
Specificity of the electrochemical DNA sensors based on terminal protection. The concentration of SA was 100 nM and the other non-specific proteins were present at concentrations of 1 μM. Error bars represent the standard deviation of the measurement (n = 3).

The specificity of this electrochemical DNA sensor was also analyzed using SWV by recording the signal intensity for various other non-specific proteins. These proteins include bovine serum albumin (BSA), thrombin, vascular endothelial growth factor (VEGF), and insulin. It can be seen from Figure 7 that the SWV signal intensities for BSA (1 μM), thrombin (1 μM), VEGF (1 μM), and insulin (1 μM) are slightly higher than that of the control group (blank group) without SA. However, the SWV signal intensity for the non-specific proteins was significantly lower than the SWV signal intensity for 100 nM SA. This indicates that these non-specific proteins have minimal effects on the hydrolysis reaction involving the digestion of M1-Biotin DNA by EXO III. This further demonstrates that the binding of SA to biotin is specific and that the specific binding of biotin and SA protects the 3′ end of M1-Biotin DNA from EXO III hydrolysis. This suggests that the terminal protection method is highly selective for the detection of small molecule–target protein interactions.

### 3.7. Anti-Interference

**Figure 8:**
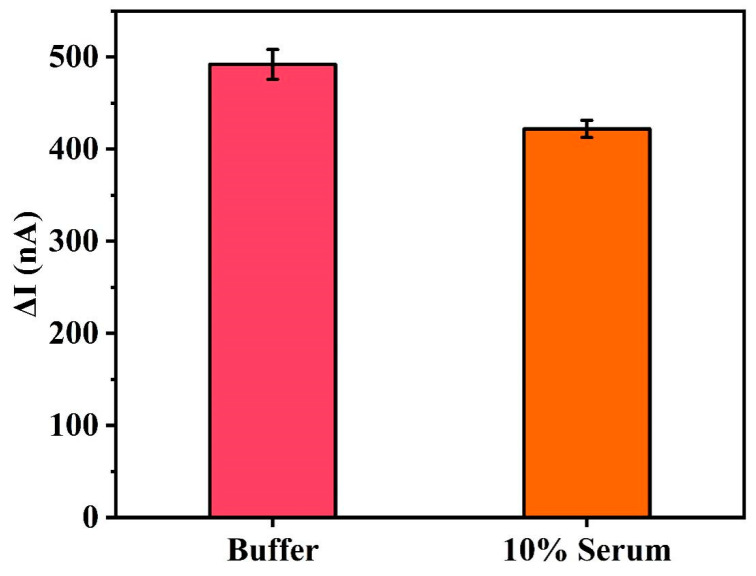
Signal difference (ΔI) for the electrochemical DNA sensor based on terminal protection challenged with target (1 μM) in buffer (20 mM Tris-HCl, 100 mM NaCl, 20 mM MgCl_2_) and complex matrices, such as 10% serum. Error bars represent the standard deviation of the measurement (n = 3).

In order to further prove the performance of the electrochemical DNA sensor based on terminal protection, the target protein (1 μM) was added to 10% serum to directly challenge the sensor in the actual sample. From Figure 8, we observe that the signal change of the sensor when incubated in buffered saline or interfering substance (10% serum) is negligible (<17%), although their composition is extremely complex and multi-component nature may reduce the sensor performance. This outstanding anti-interference is mainly due to the small molecule–protein interaction, the catalysis of exonuclease III, and the terminal protection effect.

### 3.8. Detection Ability of Other Proteins

**Figure 9:**
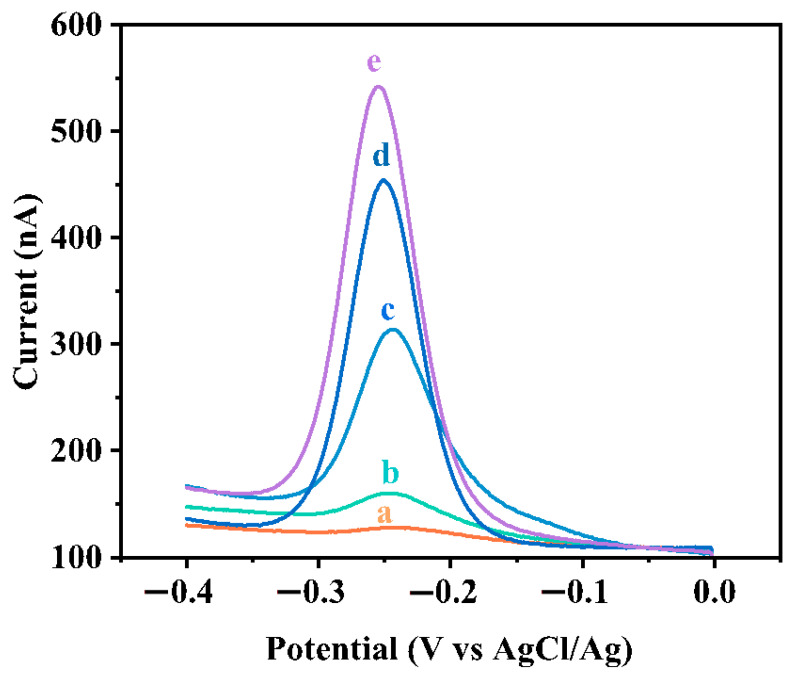
Typical SWV responses of the electrochemical DNA sensors to different concentrations of FR, from bottom to top (**a** to **e**): 0 nM, 1 nM, 50 nM, 500 nM, 1 μM in a buffer solution (10 mM Tris–HCl, 100 mM NaCl, 20 mM MgCl_2_, pH 7.4).

In order to measure the ability of this sensor to detect other protein molecules, we detected the folate receptor (FR) under optimal conditions. Before that, we changed the modified small molecule on the 3′ end of DNA to folate (FA). As shown in Figure 9, when the concentration of the detection substance is zero (curve a), only a weak signal is detected. When FR is added to the solution, a clear electrical signal can be observed. Additionally, as the FR concentration increases, the peak SWV current gradually increases, which indicates that the quantitative analysis signal response is highly dependent on the concentration. It also shows that this strategy can detect other proteins.

## 4. Conclusions

In this work, a DNA electrochemical detection platform for detecting proteins based on the terminal protection effect and the interaction of small molecule labeled DNA with proteins has been developed. We demonstrated this strategy by using a small molecule biotin and its receptor protein streptavidin (SA) as a model example. The sensor platform can detect protein molecules sensitively, accurately and quickly through the binding and protection of protein molecules to the 3′end of small-molecule-linked DNA. The biosensor obtains a wide linear range and a low detection limit. At the same time, the biosensor platform has been proven to be able to detect in the serum environment. Additionally, we detected the folate receptor (FR) to confirm that this strategy can be used to detect other proteins. It is expected that numerous other electrochemical DNA biosensors can be developed using our method, which is based on the small molecule–protein interaction strategy and terminal protection effects. Based on these preliminary studies, the method is expected to have potential clinical applications, and it is expected to detect other protein molecules by replacing the small molecule part of DNA connection.

## Figures and Tables

**Table 1:** Sequences of the oligonucleotides used in this study.

Name	Sequence (5′-3′)
B1-DNA	5′-HS(CH_2_)_6_-TTACCACTGACGCAACTTGCACTACGCAATCCACTTAG-biotin-3′
S1-DNA	5′-CTAAGTGGATTGTCACGTGGAACTACTACCAAT-MB-3′
S2-DNA	5′-AGTAGTTCCACGCTGCGTAGTGCAAGTTGCAAGTCA-MB-3′

**Table 2:** Comparison of the proposed assay with other established methods for DNA-binding protein detection.

Methods	Linear Range	Detection Limit	Refs
Fluorescence	0.5 nM to 1 μM	0.1 nM	[19]
Electrochemistry	-	0.22 nM	[31]
Electrochemistry	0.8 nM to 0.5 μM	263 pM	[32]
Electrochemistry	5 pM to 10 nM	5 pM	[33]
Electrochemistry	1 μM to 8 μM	177 nM	[34]
Electrochemistry	0.5 nM to 5 μM	18.8 pM	This work

## Data Availability

Not applicable.
